# Correction: Targeting EGFR/PI3K/AKT/mTOR signaling in lung and colon cancers: synthesis, antitumor evaluation of new 1,2,4-oxdiazoles tethered 1,2,3-triazoles

**DOI:** 10.1039/d4ra90066a

**Published:** 2024-06-05

**Authors:** Mohammed Salah Ayoup, Islam Shawki, Hamida Abdel-Hamid, Doaa A. Ghareeb, Aliaa Masoud, Marwa F. Harras, Mohamed El-Atawy, Nuha Salamah Alharbi, Magda M. F. Ismail

**Affiliations:** a Department of Chemistry, College of Science, King Faisal University Al-Ahsa 31982 Saudi Arabia mayoup@kfu.edu.sa mohammedsalahayoup@gmail.com; b Department of Chemistry, Faculty of Science, Alexandria University Alexandria Egypt mohamed.elatawi@alexu.edu.eg; c Bio-Screening and Preclinical Trial Lab, Biochemistry Department, Faculty of Science, Alexandria University Alexandria Egypt; d Department of Pharmaceutical Medicinal Chemistry and Drug Design, Faculty of Pharmacy (Girls), Al-Azhar University Cairo 11754 Egypt; e Chemistry Department, College of Science at Yanbu, Taibah University Yanbu 46423 Saudi Arabia; f Chemistry Department, College of Sciences, Taibah University Al-Madina 30002 Saudi Arabia; g Center of Excellence for Drug Preclinical Studies (CE-DPS), Pharmaceutical and Fermentation Industry Development Center, City of Scientific Research & Technological Applications (SRTA-city) New Borg El Arab Alexandria Egypt

## Abstract

Correction for ‘Targeting EGFR/PI3K/AKT/mTOR signaling in lung and colon cancers: synthesis, antitumor evaluation of new 1,2,4-oxdiazoles tethered 1,2,3-triazoles’ by Mohammed Salah Ayoup *et al.*, *RSC Adv.*, 2024, **14**, 16713–16726, https://doi.org/10.1039/D4RA02222J.

The authors regret that the one of the affiliations (affiliation *g*) was incorrectly shown in the original manuscript. The corrected list of affiliations is as shown here.

The Royal Society of Chemistry apologises for these errors and any consequent inconvenience to authors and readers.

## Supplementary Material

